# Implementation and effect of intensified case finding on diagnosis of tuberculosis in a large urban HIV clinic in Uganda: a retrospective cohort study.

**DOI:** 10.1186/1471-2458-12-674

**Published:** 2012-08-20

**Authors:** Sabine Hermans, Esther Nasuuna, Frank van Leth, Elena Byhoff, Miriam Schwarz, Andy Hoepelman, Joep Lange, Yukari C Manabe

**Affiliations:** 1 Infectious Diseases Institute, Makerere University College of Health Sciences, PO Box 22418, Kampala, Uganda; 2 Department of Global Health, Academic Medical Center, University of Amsterdam, Amsterdam Institute for Global Health and Development, Amsterdam, The Netherlands; 3 Department of Internal Medicine and Infectious Diseases, University Medical Center Utrecht, Utrecht, The Netherlands; 4 University of Pennsylvania School of Medicine, Philadelphia, PA, USA; 5 Fogarty International Clinical Research Scholars Program, Washington, WA, USA; 6 Department of Medicine, Division of Infectious Diseases, Johns Hopkins University School of Medicine, Baltimore, MD, USA

**Keywords:** Intensified case finding, Tuberculosis, Screening, HIV/AIDS, Implementation research, Resource-limited setting, Costing analysis, Yield

## Abstract

**Background:**

Increased detection of tuberculosis (TB) using intensified or active case finding (ICF) is one of the cornerstones of the Stop TB Strategy, and contrasts with passive case finding (PCF) which relies on self-reported symptoms. There is no clear guidance on implementation strategies. We implemented ICF in addition to ongoing PCF in our large urban HIV clinic in July 2010 using a twice-daily announcement screen method by a trained peer educator, asking waiting patients to self-refer to a trained peer supporter for screening of TB symptoms. We sought to determine the associated effect on TB case detection.

**Methods:**

Suspects were investigated by sputum smear, chest X-ray and ultrasound, if indicated. Routinely collected clinical and laboratory data were merged with the ICF register and TB clinic data for patients attending the clinic in 2010. We compared the yield of TB cases (defined as the prevalence of newly diagnosed TB cases in the screened population), the type of TB diagnosed and the total cost per TB case identified (in United States Dollars [USD]) for the period before and after ICF implementation.

**Results:**

Of the 20,456 patients who visited the clinic in 2010, 614 were identified as TB suspects, 220 pre-ICF and 394 post-ICF (229 via PCF and 165 via ICF). The proportion diagnosed with TB dropped from 66% to 48% (60% in suspects identified through PCF and 31% through ICF). During the post-ICF period, TB suspects identified through ICF compared to PCF identification were more likely to be female, older, on ART and to have been enrolled in HIV care for a longer duration. The yield of combined PCF and ICF screening was 1.4% pre-ICF and 1.7% post-ICF with a cost per TB case identified of 12.29 USD and 21.80 USD, respectively.

**Conclusions:**

Implementation of ICF in a large HIV clinic yielded more TB suspects and cases, but substantially increased costs and was unable to capture the majority of TB suspects who were referred for diagnosis by clinicians through PCF. The overall yield of TB cases in a mature HIV clinic was low, although targeted screening of those recently enrolled in care may increase the yield.

## Background

HIV is the most important risk factor for the development of tuberculosis (TB). People living with HIV are 21-34 times more likely to develop TB than the HIV-uninfected [1]. TB is a leading cause of death among HIV infected people with 22% of all deaths attributable to TB.

In 2004, collaborative activities to reduce the burden of TB-HIV co-infection were added to the Stop TB Strategy of the World Health Organization (WHO) [2]. These activities include provision of antiretroviral therapy (ART) and the “3Is*”*: intensified case finding (ICF), isoniazid preventive therapy (IPT) and infection control. ICF refers to regular screening of all people with or at high risk of HIV or, in congregate settings, for symptoms of TB disease followed by prompt diagnosis and treatment [3]. It is the “gatekeeper” of the “3Is” as it identifies eligible patients for IPT and aids infection control [3,4]. It differs from passive case finding (PCF) which detects TB cases among symptomatic patients presenting for diagnosis and treatment of symptoms [5].

Uganda is one of the 22 WHO TB high-burden countries with an HIV prevalence of 6% [1,6]. The case detection rate of 61% in 2010 falls short of the WHO target of 70%. In order to increase the number of HIV-positive patients diagnosed with TB, the Ugandan Ministry of Health (MoH) in October 2008 issued a mandate for all health clinics to start ICF. In collaboration with the National Tuberculosis and Leprosy Programmed (NTLP), it developed a screening form to be administered in all health care settings to aid in the detection of tuberculosis among high-risk groups (people living with HIV/AIDS, or contacts of known TB patients).

Three ways to implement ICF in our high-burden HIV clinic in Kampala were tested for feasibility in October 2009; (1) the individual screen method: every patient was screened upon entry to the waiting area; (2) the poster screen method: posters in English and Luganda (the most widely spoken local language) were placed in the waiting area and included educational bullet points about TB and TB-HIV co-infection, the ICF screening questions, and instructions to approach a designated peer supporter (an IDI patient who receives a reimbursement for provided services in the clinic) if a patient recognized any of these symptoms; and (3) the announcement screen method: a trained peer educator gave twice daily presentations in English and Luganda on TB and TB-HIV co-infection and the ICF screening questions in the waiting area, asking waiting patients to approach a trained peer supporter for screening if they harbored any of the symptoms. Each method was piloted on 2 clinic days and then compared. The first method necessitated 4 additional full-time screening staff who only managed to screen 78% of patients. Furthermore, the increased TB suspect workload was unsustainable due to the additional human resource requirements, although arguably the workload could decrease over time. The second method was not feasible as 23% of patients reported being illiterate. The third method identified a similar number of TB cases as the first method with less increase in workload. Therefore, we decided to implement ICF using the announcement screen method. In this study we set out to determine the associated effect on case detection of TB.

## Methods

### Setting

The Adult Infectious Diseases Clinic at the Infectious Diseases Institute (IDI) is a large urban HIV clinic in Kampala, Uganda, which provides outpatient HIV care to approximately 10,000 active patients. Around 600 patient visits are scheduled daily; the majority of these (85%) are scheduled monthly return visits for chronic HIV care and medication refills. The other 15% are unscheduled emergency visits, new patient visits or referrals. The HIV and TB-HIV outpatient clinics at IDI have been described in detail previously [7,8]. In brief, care is based on the Uganda national guidelines, recommending co-trimoxazole prophylaxis for all HIV-infected persons and, at the time of our study, provision of ART to all with a CD4+ T cell (CD4) count lower than 250 cells/mm^3^[9]. In 2008, an outdoor integrated TB-HIV clinic was set up to standardize the clinical management of patients suspected of or with TB-HIV co-infection [8]. It serves around 25 to 45 patients daily and is staffed by dedicated doctors and nurses trained in TB and HIV co-management. The TB-HIV clinic provides all care for both TB and HIV (including provision of ART, diagnosis and treatment of any other opportunistic infections).

Before the introduction of ICF, patients suspected of having TB were identified via PCF and sent to the TB-HIV clinic by the triage nurses in the general HIV outpatient clinic or by the doctors from their consultation rooms. All TB suspects were seen in the TB-HIV clinic for diagnosis and treatment. TB investigations including sputum smear microscopy, chest radiology, abdominal ultrasonography, lymph node biopsy and fine needle aspiration for microscopy were used. Mycobacterial cultures were not routinely available due to cost. Diagnosis was based on these investigations and occasionally on clinical presentation only. TB was treated according to the National TB and Leprosy Program (NTLP) guidelines [10].

### Implementation of ICF

The MoH ICF screening form consisted of a set of five TB screening questions: cough (for more than 2 weeks), hemoptysis, fevers (for more than 3 weeks), excessive night sweats (for more than 3 weeks) and weight loss (more than 3 kilograms in one month). A person with any of these symptoms was considered a TB suspect and should be investigated for TB. This form was to be used for TB screening in HIV care settings at each clinic visit and was to be administered by trained personnel, either a health care professional or lay person, at the health facility. Per the MoH recommendations, all patients visiting the HIV clinic were targets of ICF screening.

In July 2010, we implemented ICF clinic-wide using the announcement screen method. Two trained peer supporters were designated and trained to give twice daily presentations on TB and TB-HIV co-infection and the ICF screening questions (in English and Luganda) in the general clinic waiting area, asking waiting patients to approach one of them if they had any of the described symptoms. They were also to screen the patients presenting themselves to them afterwards and to record all who reported one or more of the symptoms on the screening form in a register. They referred these TB suspects to the outdoor TB-HIV clinic directly, where they were further investigated for TB according to the clinic guidelines. Their scheduled HIV clinic visit was also completed in the TB-HIV clinic. In addition to ICF, TB suspects continued to be identified by PCF during their encounter with the HIV clinic staff at their scheduled visit.

### Study design, selection criteria and definitions

**Figure 1:**
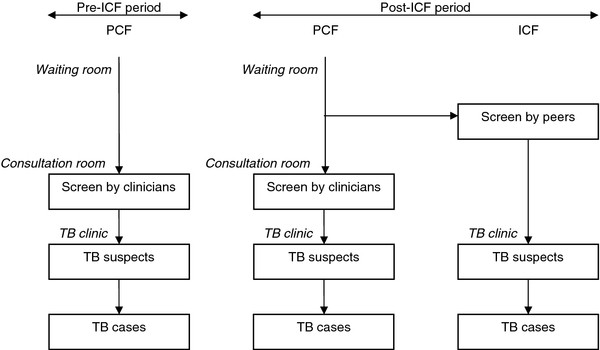
**Study overview: screening for TB before and after implementation of ICF.** Note: *ICF*, intensified case finding; *PCF*, passive case finding; *TB*, tuberculosis.

This study used a retrospective cohort study design in which we compared two periods: six months before ICF, January to June 2010 (pre-ICF), and 6 months after ICF implementation, July to December 2010 (post-ICF) (Figure 1). All patients with an IDI clinic visit during 2010 were eligible for inclusion in our analysis. Only one clinic visit per patient per study period was used to calculate the screened population. The median number of repeat visits per patient per period was also determined. A TB suspect was defined as a patient who was referred to the TB clinic for TB investigation, irrespective of their method of identification (PCF or ICF). Identification via PCF or ICF in the post-ICF period was determined by the ICF register in which all suspects identified via ICF were recorded (see above). A TB diagnosis was defined as having received a TB diagnosis from the TB-HIV clinic medical officer, based on the procedures explained above. TB treatment was defined as having been started on TB treatment.

All patients who screened positive for TB by the peer supporter, but who did not classify as TB suspects on arrival at the TB clinic (n = 50), who never presented to the TB clinic for further work-up of TB (n = 32), or for whom no TB work-up data was available and whose files were unavailable for review (n = 56) were excluded. We also excluded patients transferred from other health centers with an existing TB diagnosis and patients who were already on TB treatment.

### Study outcomes

The primary outcome measure was the yield of newly diagnosed TB patients identified (defined as the prevalence of newly diagnosed TB cases in the screened population). Secondary outcomes included the type of TB diagnosed and the total cost per TB case identified. All outcomes were compared for the period before and after ICF implementation.

### Data collection and ethics statement

Routinely collected data on clinical parameters, ART and adherence, WHO stage, toxicities and opportunistic infections of all IDI patients were entered into a database, to which laboratory data were added electronically. We extracted data from this database, from the ICF register (of all patients who screened positive by the trained ICF peer supporters), the electronic TB-HIV clinic database (which contained routinely collected data on TB symptoms, diagnosis, treatment and follow-up at each TB clinic visit), and the NTLP TB register (of all patients on TB treatment at IDI). We merged these data to arrive at a complete dataset of all patients who had visited IDI in 2010, and which included detailed information on those screened for TB. We performed chart reviews to validate any inconsistent data between these data sets. Patients lost to follow-up were recorded at each stage of the diagnostic process from screening to treatment. This study was performed as part of regular clinic monitoring and evaluation for continuous quality improvement that was approved by the Institutional Review Boards of IDI, Makerere University College of Health Sciences and the Uganda National Council for Science and Technology.

### Data analysis

The absolute numbers and proportions of suspects, patients having undergone TB investigations, patients with a final diagnosis (TB yes/no) and patients treated for TB in both periods were compared. Comparisons were done by period, by method of identification and by time since registration at the IDI (classified as enrolment into care in the 3 months prior to their becoming a TB suspect or after). Baseline characteristics were compared using Chi-squared, two-tailed student t-tests and, where appropriate, two-sample Wilcoxon rank-sum tests. Ninety-five percent confidence intervals (CI) were used and *P*-values ≤0.05 were considered to be statistically significant. Analyses were conducted using STATA version SE 11.1 (College Station, Texas, USA).

### Cost inputs and analysis

We assessed all costs to perform the screening and diagnosis of TB suspects, including the costs for investigations and for the peer supporters performing the screening. The peer supporters had no other clinic responsibilities than the TB screening programmed. Costs were the actual price paid for these services in 2010. As the real number of investigations performed was not captured in our database, we estimated these based on current clinic standard operating procedures and on the proportion of extrapulmonary TB diagnosed in the pre-ICF and post-ICF periods (subdivided by suspects identified via PCF and via ICF). We assumed that all TB suspects underwent a chest X-ray and sputum smear analysis (2 smears). We assumed that half of the investigations for extrapulmonary TB consisted of a lymph node aspirate and the other half of an abdominal ultrasound. Other investigations such as a lymph node biopsy and pleural fluid analysis were performed very infrequently, and were therefore not included in the analysis. As the TB clinic and staff remained the same in the pre- and post-ICF periods, these costs were not included, as were costs for routine HIV care, such as laboratory investigations. All costs were totaled and divided by the number of TB cases identified to arrive at the cost per TB case.

## Results

### Baseline characteristics

A total of 9,931 patients visited the IDI in the six months pre-ICF and 10,525 in the six months post-ICF implementation. These patients visited the clinic a mean of 4.7 times (standard deviation [SD], 1.9) per person in the pre-ICF period and 4.8 (SD, 2.0) times in the post-ICF period. More women than men visited the clinic (68% in both periods), and 74% and 72% were on ART at their first visit during the pre- or post-ICF period, respectively. In the pre-ICF period, patients had been registered at the IDI for a median of 1441 (interquartile range [IQR], 563, 1769) days before their first clinic visit during those six months; in the post-ICF period this was a median of 1536 (IQR 511, 1930) days.

A total of 614 patients were suspected of having TB over both periods studied: 220 (2.2%) pre-ICF and 394 (3.7%) post-ICF, of which in the latter 165 (41.9%) were identified via ICF and 229 (58.1%) via PCF. Overall, 356 (58%) were women and 217 (35%) were on ART. See Table 1 for the baseline characteristics of all three groups. Suspects identified via PCF in both periods seemed to be relatively similar, but suspects identified through ICF as opposed to through PCF during the post-ICF period were more likely to be female, older, on ART and to have been registered at the IDI for a longer duration.

**Table 1:** Patient characteristics

		**Pre-ICF**	**Post-ICF**	**Post-ICF**	***P*-value**	***P*-value**
**Characteristic**		**PCF suspects**	**PCF suspects**	**ICF suspects**	**PCF pre vs PCF post**	**PCF post vs ICF post**
		**N = 220 (100%)**	**N = 229 (58%)**	**N = 165 (42%)**		
Sex (n [%])	Male	112 (51)	95 (41)	51 (31)	**0.045**	**0.032**
	Female	108 (49)	134 (59)	114 (69)		
Age (years, mean [SD])		35 (9.2)	36 (10.1)	40 (10.0)	0.300	**<0.001**
ART (n [%])*	Yes	68 (31)	75 (33)	74 (45)	0.411	**0.007**
	No	149 (68)	147 (64)	91 (55)		
Time at IDI since registration (days, median [IQR])		94 (4, 1005)	57 (1, 1437)	1015 (128, 1830)	0.696	**<0.001**
Symptoms (n [%])*	Pulmonary	164 (75)	187 (82)	138 (84)	**0.033**	**0.011**
	Only B-symptoms	20 (9)	23 (10)	5 (3)		
	Missing	36 (16)	19 (8)	22 (13)		
		**TB patients**	**TB patients**	**TB patients**		
		**N = 139 (63%)**	**N = 131 (57%)**	**N = 49 (30%)**		
Type of TB (n [%])	Smear positive	60 (43)	39 (30)	19 (39)	0.051	0.824
	Smear negative	20 (14)	28 (21)	12 (24)		
	Extrapulmonary	54 (39)	51 (39)	15 (31)		
	Missing	5 (4)	13 (10)	3 (6)		

*Data on ART use was not available for 10 patients (3 pre-ICF and 7 post-ICF of which all PCF).

Note: *ART*, antiretroviral therapy; *ICF*, Intensified Case Finding; *IDI*, Infectious Diseases Institute; *IQR*, interquartile range; *PCF*, passive case finding; *SD*, standard deviation; *B-symptoms*: fevers or night sweats for more than 3 weeks and/or weight loss (>3 kilograms in one month); *vs*, versus.

### Outcomes before and after ICF

**Figure 2:**
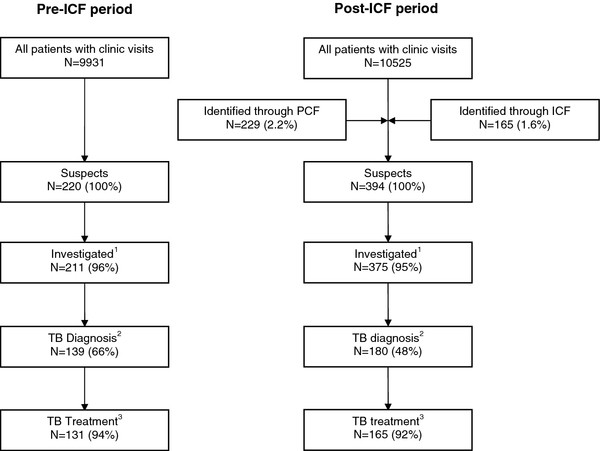
**Patient flow before and after ICF Implementation.** This figure shows the flow of patients in the two periods assessed in this study. ^1^Pre-ICF, 9 were not investigated (4%): 3 LFU, 1 died, 5 charts missing. Post-ICF, 14 were not investigated (3 LFU, 1 psychotic, 1 LFU to general clinic, 9 missing) and 5 charts were missing. ^2^Pre-ICF, in 66 no TB was found (31%); 6 diagnoses were missing (3 LFU, 1 died, 2 charts missing). Post-ICF, no TB was found in 185 (49%); 10 diagnoses were missing (4 LFU, 2 died, 1 LFU to general clinic, 3 charts missing). ^3^Pre-ICF, 8 were not treated for TB (1 LFU, 2 died, 5 missing). Post-ICF, 15 (8%) were not treated for TB (2 LFU, 5 died, 8 charts missing). Note: *ICF*, intensified case finding; *LFU*, loss to follow-up; *TB*, tuberculosis.

Absolute numbers of suspects, suspects investigated for TB, diagnosed with and treated for TB all increased after ICF implementation (Figure 2). The number of suspects almost doubled from 220 to 394. The proportion of suspects investigated for TB remained high at 96% in the pre-ICF period and 95% in the post-ICF period. Of those, the proportion diagnosed with TB dropped from 66% in the pre-PCF to 48% in the post-ICF period. In the post-ICF period, 49 out of 158 (31%) of investigated ICF suspects and 131 out of 217 (60%) investigated PCF suspects were diagnosed with TB.

Suspects who had enrolled into care in the 3 months prior to their becoming a TB suspect (n = 261 or 43% of all suspects) were more likely to be diagnosed with TB than those who had been at the IDI for more than 3 months (63% versus 44%, *P* < 0.001). Post-ICF, these newly enrolled suspects primarily presented via PCF (118 out of 152 [78%]) and less via ICF (34 out of 152 [22%]). There was no difference in the type of TB diagnosed between the suspects identified in the pre- and post-ICF period, or between the methods of their identification (Table 1).

### Yield of ICF

The yield of screening was 1.4% (139 of 9931) in the pre-ICF period (only PCF) and 1.7% (180 out of 10525) in the post-ICF period (ICF and PCF combined), or a prevalence of 1,400/100,000 and 1,700/100,000, respectively.

### Costs

By adding up all costs involved with the screening and the diagnosis of TB suspects before and after ICF implementation and then dividing this amount by the numbers of TB cases, we found that the cost per TB case found almost doubled from 12.29 USD pre-ICF to 21.80 USD post-ICF (Table 2). The greatest cost difference was incurred by the increase in numbers of investigations (sputum smears and chest X-rays) and the additional cost of the screening staff. Costs increased by 130% and TB cases identified by 30%.

**Table 2:** Costs incurred in the pre- and post-ICF periods

**Item**	**Unit cost (UGX)**^**1**^	**Unit cost (USD)**^**1**^	**Pre-ICF (n = 220)**	**Post-ICF (n = 394)**
Sputum smear (100%)^2^	10,000	4.32	949.42	1700.33
Chest X-ray (100%)	5,000	2.16	474.71	850.17
Ultrasound (20%)	10,000^3^	4.32	189.89	340.07
Lymph node aspirate (20%)	5,000	2.16	94.94	170.03
Peer supporter	80,000 per week x 25 weeks	34.52 per week	NA	863.11
**Total costs**			**1708.96**	**3923.71**
**Cost per TB case**			**12.29**	**21.80**

^1^Based on exchange rate 1000 UGX = 0.43156 USD (1/7/2010).

^2^Consisting of 2 sputum samples per TB suspect.

^3^Of which patients contribute 50% out of their own pocket.

Note: *ICF*, Intensified Case Finding; *NA*, not applicable; *PCF*, passive case finding; *TB*, tuberculosis; *USD*, United States Dollars.

## Discussion

We found that implementation of ICF by the announcement screen method in a large HIV clinic led to a 76% increase in the number of TB suspects identified, with only a 30% increase in newly diagnosed TB cases. PCF during the post-ICF period continued to identify a similar number of TB suspects compared with the pre-ICF period, even though ICF was performed at an earlier stage of the clinic visit than PCF. Different subsets of patients were identified through the two parallel screening methods.

The reported yield of new TB cases identified by ICF varies greatly, depending on the HIV prevalence in the population at risk studied, the country-specific TB prevalence and the method of screening used (symptom-based or not). According to a recent meta-analysis of studies of ICF in HIV-infected people in resource-limited settings, it ranged from 2.2% in contact-tracing exercises to 8.2% in medical and antiretroviral clinics [11]. Factors associated with a higher yield of ICF included the screening of all patients regardless of symptoms, and ICF in countries with higher TB prevalence. Screening strategies also vary widely and usually involve a set of questions. Absence of current cough, night sweats, weight loss and fever was associated with a low probability of active TB disease [12], although this was dependent on the prevalence of TB in the screened population with the negative predictive value decreasing from 99.7% to 92.3% with an increase in TB prevalence from 1% to 20%.

The yield of our symptom-based screening method was lower: at 1.4% and 1.7% of the active attending patient population in the pre-ICF and post-ICF period, respectively. In Uganda, prevalence estimates of newly diagnosed TB identified by symptom-based ICF screening programmes vary between 7.2% in a rural cohort of ART initiators, 3.6% in a rural cohort of patients screened for ART eligibility and 1.4% among new patients enrolling in an HIV clinic in the same hospital complex [13-15]. Our study population represents the population of a “mature” HIV clinic, with the majority of patients on ART and therefore less likely to have unrecognized TB. Also, regular TB screening via PCF may have already reduced the burden of infectious patients and thus the nosocomial transmission of TB, leading to a reduced TB incidence in our patient population. Other explanations for the low yield found in our study include an inefficient method of screening with missing of TB suspects (possibly also due to stigma, evidenced by the large proportion of TB suspects identified by PCF after ICF had been implemented), or a substandard laboratory performance, although regular external quality control has not shown this to be the case. The contribution of missed smear-negative, culture-positive TB is unclear: we diagnosed smear-negative pulmonary TB among 12% of coughing TB suspects in both the pre- and post-ICF period, which was lower than an estimated 19% smear-negative, culture-positive TB diagnosed among IDI pulmonary TB suspects in an earlier study (unpublished data from [16]). The effect of routine use of TB culture or Xpert MTB/RIF in confirming these empirically treated TB cases should be investigated, as well as the cost-effectiveness of such strategies.

We attempted to implement ICF in a methodical and feasible way using a method based on announcements. The individual screening method would possibly have led to identification of more suspects, but at a greater cost, both monetary and human resource. We show that this method is feasible, but less effective than PCF. The population of TB suspects identified by this method of ICF was different from the one identified through ongoing PCF in the same period, as evidenced by their differing baseline characteristics and the higher TB suspect to case ratio in ICF versus PCF suspects. This could have been due to inadequate screening by the peer supporters, or to a higher likelihood of more “experienced” patients presenting themselves for screening. This correlates with our finding that only one-fifth of TB suspects who were newly enrolled in HIV care were identified through ICF. Conversely, considering that the suspects identified through PCF in both periods were relatively similar but differed from those of the suspects identified through ICF, ICF might have identified an additional subset of TB patients which otherwise would have remained undiagnosed and therefore infectious. Our results cannot confirm or exclude this possibility, however. Additionally, stigma attached to TB may result in the patient presenting to the clinician when being asked the screening questions rather than approaching the peer supporter in the waiting area. Incomprehension of the message may also be a cause; either due to a language barrier or because of the manner in which the message is delivered. Our ICF method was very dependent on how the person charged with giving the announcements did this, and how he/she emphasized the importance of knowing one’s “TB status”. The individual screening method may arguably have fewer of these problems. Lastly, the announcements could possibly have made PCF more effective, by sensitizing patients and making them more likely to volunteer their symptoms during their clinician visit.

Costs per TB case identified rose from 12 to 22 USD after implementation of ICF using the announcement screen method. The increase was mainly due to the higher suspect to case ratio and to the cost of the peer supporters performing the screening. These were relative costs, comparing costs pre- and post-ICF implementation. Absolute costs of screening programmes are highly dependent on the work-up of TB suspects and comparisons across settings and countries are therefore difficult to make. We lacked both cost and clinical inputs to do a formal cost-effectiveness analysis. Our analysis was simple in nature and therefore did not take staff time or treatment costs into account. The TB-HIV clinic staff including the peer supporters would only have helped out in the regular ART clinic on an ad-hoc basis. A formal cost-effectiveness analysis is needed to inform practice and should include the effect of ICF on TB transmission. The cost-effectiveness of screening of the whole population regardless of (the duration of) symptoms, as proposed by some [11,17], would have to be established. This would not be feasible in our clinic without a comprehensive restructuring of the health care delivery at IDI and without a significant increase in resources for personnel and investigations.

Our findings raise the question how best to optimize ICF in a setting like ours. As the majority of TB cases were found among patients newly enrolled into HIV care, it seems advisable to screen that part of the patient population systematically, possibly using an individual screening method. For patients in care for a longer period of time, the optimal screening strategy is less obvious. As clinicians are less likely to think of TB in stable patients, there is a need for some form of additional screening, especially if it would sensitize the patient to report TB-symptoms during the formal encounter with the HIV clinician. Targeted announcements such as in our study might work for this category of patients. Optimal screening frequencies should be established, for example by comparing a strategy of screening at each clinic visit with 3-monthly, 6-monthly or even yearly TB screening.

Noteworthy was the high retention rate of TB suspects: 95% and 96% underwent investigations. This was substantially higher than in a similar screening programmed in Swaziland, where this was only 53% [18].

Limitations of our study were the retrospective study design and the use of routinely collected data with missing data as a result. The 50 excluded patients who were identified by ICF but did not qualify as a TB suspect on arrival at the clinic, added to the increase in workload after ICF implementation. Some of these were misidentified by the peer supporters, highlighting the need for continuous retraining of lay health care workers. Others purposefully reported TB symptoms at screening to ensure being seen by a clinician in the TB-HIV clinic. Interestingly, 32 patients were identified as TB suspects by the peer supporter but were never seen in the TB clinic, possibly due to long waiting times or to stigma associated with TB. The potential TB suspects for whom no data was available (n = 56) possibly belong to the same category. Both point towards a possible systems issue absorbing these patients in the TB-HIV clinic. Although we tried to highlight differences in study population, changes due to time-trends are a limitation of the before-after study design. We feel that the limited period of time in which the study was conducted was unlikely to have a major impact on the results.

Sputum samples were investigated by light microscopy using Ziehl-Neelsen staining in the pre-ICF period, while an in-house LED-based fluorescence microscopy was established at the start of the post-ICF period. However, we do not believe the 10% sensitivity difference to have influenced our case-detection in both periods greatly [19]. We made assumptions of the numbers of investigations ordered for the costing analysis; however, as the investigations did not differ hugely in price, we believe that using the actual numbers would not change our estimate markedly. We also assumed a flat cost of 5,000 UGX (2.16 USD) for sputum smear microscopy, while the in-house test implemented in the post-ICF period was cheaper (true costs were not available). Our costing analysis was therefore conservative.

Lastly, this method of ICF implementation would not have been possible in a large clinic without resources for the additional lay health care workers and investigations. The IDI has more available tests for diagnosis and better follow-up of the patients compared to most health care settings in sub-Saharan Africa. Also, the on-site fluorescence microscopy services could handle the additional sputum smears in a timely fashion. For ICF to be implemented countrywide in Uganda and in other resource limited settings, there would have to be a scale-up of resources in order to train health care and lay workers and to equip the laboratory systems to handle the extra workload.

## Conclusions

Implementation of ICF in a large, “mature” HIV clinic using an announcement screen method yielded more TB suspects and cases, but substantially increased costs and was unable to capture the majority of TB suspects referred for diagnosis, potentially diluting an effect on infection control. The overall yield of TB cases in a mature HIV clinic was low, raising the question whether ICF is cost-effective and affordable in such a setting. Research into the optimization of ICF is needed, including targeted screening strategies for those recently enrolled into care.

## Competing interests

The authors declare that they have no competing interests.

## Author’s contributions

Conceived and designed the experiments: SH, EN, FvL, EB, MS and YM. Analyzed the data: SH and FvL. Wrote the manuscript: SH, EN, FvL, EB, MS, AH, JL and YM. ICMJE criteria for authorship read and met: SH, EN, FvL, EB, MS, AH, JL and YM. Agree with the manuscript’s results and conclusions: SH, EN, FvL, EB, MS, AH, JL and YM. Wrote the first draft: SH and EN. All authors read and approved the final manuscript.

## Pre-publication history

The pre-publication history for this paper can be accessed here:

http://www.biomedcentral.com/1471-2458/12/674/prepub
